# Genetically Encoded Fluorescent Probe for Detection of Heme-Induced Conformational Changes in Cytochrome c

**DOI:** 10.3390/bios13090890

**Published:** 2023-09-18

**Authors:** Mehmet Yunus Genceroglu, Cansu Cavdar, Selen Manioglu, Halil Bayraktar

**Affiliations:** 1 Department of Molecular Biology and Genetics, Istanbul Technical University, Istanbul 34467, Turkey; 2 Biomedical Science and Engineering Program, Koç University, Istanbul 34450, Turkey

**Keywords:** cytochrome c, cytochrome c heme lyase, heme, FRET, fluorescence, protein folding

## Abstract

Cytochrome c (Cytc) is a key redox protein for energy metabolism and apoptosis in cells. The activation of Cytc is composed of several steps, including its transfer to the mitochondrial membrane, binding to cytochrome c heme lyase (CCHL) and covalent attachment to heme. The spectroscopic methods are often applied to study the structural changes of Cytc. However, they require the isolation of Cytc from cells and have limited availability under physiological conditions. Despite recent studies to elucidate the tightly regulated folding mechanism of Cytc, the role of these events and their association with different conformational states remain elusive. Here, we provide a genetically encoded fluorescence method that allows monitoring of the conformational changes of Cytc upon binding to heme and CCHL. Cerulean and Venus fluorescent proteins attached at the N and C terminals of Cytc can be used to determine its unfolded, intermediate, and native states by measuring FRET amplitude. We found that the noncovalent interaction of heme in the absence of CCHL induced a shift in the FRET signal, indicating the formation of a partially folded state. The higher concentration of heme and coexpression of CCHL gave rise to the recovery of Cytc native structure. We also found that Cytc was weakly associated with CCHL in the absence of heme. As a result, a FRET-based fluorescence approach was demonstrated to elucidate the mechanism of heme-induced Cytc conformational changes with spatiotemporal resolution and can be applied to study its interaction with small molecules and other protein partners in living cells.

## 1. Introduction

The metalloprotein c-type cytochrome (Cytc) plays a crucial role in regulating many key cellular processes, including energy metabolism, electron transfer, transmembrane potential, and initiating apoptosis in eukaryotic cells [1,2,3,4,5,6]. The delocalization from the membrane, phosphorylation, and binding to Apaf1 activate mitochondrial signaling and initiate caspase activity [7,8,9,10,11]. The disruption of these metabolic pathways affects cell fate and results in changes in normal cellular process [12]. The conserved mitochondrial biogenesis of Cytc requires many key steps, including the transport of polypeptide across the mitochondrial outer membrane, attachment of the heme (iron-protoporphyrin IX) center, its release, and refolding into a catalytically active state [13,14,15,16,17]. Unlike other heme-containing proteins, heme is covalently attached to the protein backbone, which is catalyzed by the cytochrome c heme lyase (CCHL) [18,19]. Cytc has a universally conserved peptide motif of Cys-X-X-Cys-His (CXXCH), where X represents different amino acids. Two thiol groups at Cys14 and Cys17 positions are covalently attached to heme at the inner mitochondrial membrane. Heme is not only required for enzymatic activity but also regulates the folding that induces the formation of holoCytc. The coordination of Met80 and His18 residues with heme is crucial for the formation of a catalytically active state and the regulation of electron transfer in mitochondria [20].

The folding of Cytc from apoform requires a conserved mechanism that relies on both the heme cofactor and CCHL [14,21,22,23]. Many changes in the secondary structure of Cytc were observed during the maturation of Cytc [24,25,26,27]. The folding of the unstructured peptide chain of Cytc to its native form occurs via intermediate states [28,29,30]. Due to the covalent attachment of heme to Cytc, the folding mechanism is more complex than other metalloproteins and involves many steps and interactions, including sequential binding to both heme and CCHL, coordination with heme, and refolding into the native state [31,32,33,34]. Since the insertion of heme and folding are tightly regulated, the lack of cooperativity between Cytc and CCHL is associated with the formation of inactive Cytc. The aggregation is also attributed to the formation of abnormal signaling in cells and associated different diseases described in previous studies [35]. Moreover, mutations and deletions, causing slow maturation and Cytc misfolding, can change the redox potential; therefore, they have been implicated in various disorders [36,37,38,39]. The existence of different catalytic states with a large range of oxidative potential may be the result of the presence of various folding states and heme coordination of Cytc that have to be characterized in detail [6]. Therefore, we can understand the role of different structural states in the many diverse functions of Cytc in cells.

Previous studies suggested that unique spectral properties of Cytc that catalyze different enzymatic reactions can arise due to its elastic structure, where the coordination of amino acids with heme is dynamically altered in the cells [40]. The changes in protein conformation, axial orientation, and heme geometry within Cytc may result in different states that vary the solvent exposure and access to heme iron; therefore, a large range of redox potential arises to metabolize different biochemical reactions [41]. Many studies have been conducted to investigate the mechanism of CCHL catalyst heme insertion into Cytc changes in heme coordination by using various characterization methods [42]. The most common approach to study the attachment of heme to Cytc is the use of an in vitro reconstitution method that requires the disruption of cells and the isolation of proteins [43]. The conformational changes of Cytc and the role of CCHL were investigated with different spectroscopy methods. Mass spectroscopy has been applied to determine the number of hemes attached to the Cytc [44]. The Cryo-EM method was recently used to determine the different conformational states of Cytc synthase and its role in the maturation of Cytc [43,45]. The changes in the position of the distal axial ligand and heme coordination have been determined by the changes in the Soret band detected with absorption spectroscopy [46]. Alternative indirect methods such as circular dichroism [47], X-ray spectroscopy [48], IR [49,50], H/D exchange [24,51,52], NMR [53,54,55,56], FTIR [57], and isothermal calorimetry [58] have been applied to study Cytc folding and its interaction with CCHL and heme [59]. While the use of these approaches are the most promising to study heme insertion and Cytc maturation, they have some limitations in monitoring these dynamics within cells. The primary drawbacks include the lack of sensitivity, a narrow dynamic range, and the requirement for purified proteins. Consequently, they cannot be used for detailed characterization and to comprehensively address the issues of heme recognition, Cytc maturation, and its variants within cellular contexts. The presence of different conformation states and their association with diverse sets of functions are not clearly understood due to the limitations of methods for studying Cytc structural changes at spatial and temporal resolution. At present, we lack an ideal imaging system for monitoring the intracellular dynamics of Cytc in cells. There is increasing interest in developing methods to elucidate the mechanism of CCHL-catalyzed heme insertion to Cytc in physiological conditions. To better understand the formation of the Cytc-CCHL heterodimer complex with heme and the relevance of previously proposed mechanisms in vivo, new assays can enable the detection of their interactions and understand the role of various perturbations such as point mutations and deletions.

The use of the fluorescence resonance energy transfer (FRET) method allows the detection of conformational changes at high spatial and temporal resolution [60,61,62,63,64,65,66,67]. Briefly, genetically encoded fluorescence probes, known as donor and acceptor groups, having a spectral overlap were attached to the protein of interest, and their coupling through distance change was closely monitored by using dual-color fluorescence imaging methods. If the rearrangement of these groups occurs in the range of 10 nm or less, these donor and acceptor groups attached to the protein terminals can be directly used to measure the distance change proportional to the FRET amplitude change. Therefore, it provides a quantitative tool to measure the proximity of pairs and determine folding events. Cyan fluorescence protein (CFP)–yellow fluorescent protein (YFP) or its brightest analog of Cerulean (Cer)–Venus (Ven) pairs are the primary backbones to label the proteins described in previous studies [68,69,70]. After they are attached to the N and C regions of the protein, distance change affecting the brightness of the acceptor can be quantitively measured upon excitation of the donor [71]. We previously found that Cytc redox state could be monitored with the photochromic energy transfer method by measuring the spectral shifts that modulate the emission of the enhanced yellow fluorescent protein attached to the C terminal of Cytc [72]. To gain a more direct understanding of Cytc maturation, the development of genetically encoded methods has been anticipated to reveal these dynamics within the cell. These approaches have proven useful for real-time monitoring of intracellular protein dynamics. They allow for a real-time examination of the heme-induced conformational changes of Cytc. Although the mechanism of CCHL catalyst heme insertion into Cytc is studied by using purified proteins and different characterization methods, the relevance of these proposed mechanisms can be tested in living cells by using genetically encoded probes.

We, here, demonstrate a FRET method to study the Cytc conformational changes and its dependence on heme binding in the presence of CCHL in living cells. To elucidate the mechanism of Cytc folding and overcome the limitation of current methods, we prepared a construct where Cytc gene was inserted into the Cer–Ven (C–V) backbone and transiently expressed in the cells. We studied its interaction with heme and CCHL where the fluorescence signal was acquired by using the three-channel fluorescence imaging method. The adaptive tracking method demonstrated previously to track single cells was applied to acquire the fluorescence signal changes [73]. The signal intensities for different conformational states were determined by computing the corrected FRET (netFRET) and normalized FRET (normFRET) magnitudes. Two internal standards, C17V (a linker with 17 amino acids) and C–V coexpressed in cells, were used as a reference value to compare our results. In the absence of both heme and heme lyase, the occurrence of undetectably small energy transfer was a clear indicator of the unfolded conformational state of Cytc due to the large separation of donor and acceptor groups. After heme addition in the absence of CCHL, an increase in normFRET amplitude demonstrated a partial conformational change of apoCytc that induced a large decrease in the distance between Cer and Ven. The detected intermediate state indicates that Cytc undergoes a conformation change, although covalent attachment was not present between heme and the Cytc backbone. After the expression of CCHL in cells, the magnitude of normFRET signal reached its highest level, demonstrating that the pairs remained at the closest distance. The large shift at normFRET distribution was an indicator of folded Cytc. In the absence of heme, the expression of CCHL exhibited a minimal FRET signal change, indicating a weak association with Cytc. A small increase in normFRET may be the indicator of the second intermediate state where local folding event occurs in Cytc. However, the heme binding has a significant effect on Cytc folding compared to its interaction with CCHL. The distance between terminals at different states was also determined by computing the normalized magnitude of FRET, which was compared with reference values obtained from C17V. As a result, a fluorescence-based assay, including the detailed analysis of normFRET magnitude, was demonstrated to study the conformational changes in living cells. Our methods provide real-time monitoring of Cytc conformational states with a high dynamic range, which also makes them well suited for high-throughput assays. In contrast to other studies, it permits the study of Cytc folding in physiological conditions and provides a sensitive technique to determine its interaction with CCHL and heme.

## 2. Materials and Methods

### 2.1. Reagents

Dulbecco’s Modified Eagle Medium (DMEM) sterile filtered, fetal bovine serum (FBS), and penicillin–streptomycin were obtained from Sigma Aldrich (St. Louis, MO, USA). HyClone Dulbecco’s phosphate buffer saline (DPBS)/modified (1X) with calcium, magnesium and HyClone PBS (1X) without calcium, magnesium were obtained from Fisher Scientific (Waltham, MA, USA). 0.05% Trypsin-EDTA (1X) was obtained from ThermoFisher (Waltham, MA, USA). Chemicals were used as received without any further purification. DH5a and BL21 (DE3) *E. coli* cells were purchased from Invitrogen (Carlsbad, CA, USA). The pBTR-1 (Amp) plasmid containing the iso-Cyt *c* gene and the yeast Cyt *c* heme lyase gene was kindly provided by Bruce E. Bowler of the University of Montana, Missoula, and Montana. C17V containing plasmid having CMV promotor was kindly provided by Nathan Lack of Vancouver Prostate Centre, Vancouver, and British Columbia. To insert Cytc into C17V plasmid, Cytc was amplified using the forward primer (5′-AAAACTCGAGTGACTGAATTCAAGGCCGGT-3′) and reverse primer (5′-AAAAAAGCTTCACTGGCTTTTTTCAAGTAG-3′) and was cloned into the XhoI and HindIII restriction site of C17V backbone carrying expression vector via standard cloning methods. The resulting plasmid was named C-apoCytc-V. C17V fusion construct with a linker length of 17 amino acids was used as an FRET standard in our experiments. Cer and Ven cotransfected cells were used as a negative control of FRET. The sequence of the plasmid was verified (Figure S1) at the sequencing center of Macrogen (Amsterdam, The Netherlands).

### 2.2. The Preparation of Cytc Expressing in the Cells

The complete DMEM containing 10% FBS and 1% penicillin/streptomycin was used for the growth of HeLaS3 or RPE cells in fibronectin-coated culture plates. They were placed in a 37 °C, 5% CO_2_ incubator and passaged every two days to avoid any overgrowth. When the cell confluency reached 70%, they were harvested and washed in falcon tubes and were transferred to 6- or 12-well plates with a concentration of 0.1 × 10^6^ cells/plate. After transferring the cells to well plates, they reached 50% confluency at 18 h of incubation and were ready to be transfected. DNA, OptiMEM, and transfection reagent were mixed according to the manufacturer’s protocol. For each cell, 500 ng C-apoCytc-V plasmid, 100 µL OptiMEM (at reduced serum media), and 1.5 µL of Fugene HD were mixed and incubated for 30 min at room temperature in an Eppendorf tube before being transferred to the wells. The expression was monitored by using a fluorescence microscope, and image acquisition was initiated 6 h after the transfection. The same protocol was used to transfect for other plasmids, named CCHL, Cer, Ven, and C17V. For inhibition of heme biosynthesis and to reduce its levels, the cells were treated with 0.5 mM succinylacetone (from 100 mM stock). After 6 h, the cells were transferred to an inverted fluorescence microscope to record videos. The medium without heme was used in all experiments, and heme was gradually added to the solution.

### 2.3. Spectroscopic Characterization of Cytc Constructs

Both C-apoCytc-V and C-holoCytc-V, prepared using pBTR-1 plasmid, were expressed in BL21 (DE3) cells and purified using Ni-NTA column; then, they were diluted and transferred to the transparent glass cuvette. For steady-state fluorescence measurements, the emission signal was recorded by a fluorescence spectrophotometer (Shimadzu (Kyoto, Japan) RF-5301, DE) using a 150-W Xenon lamp. A wavelength of 430 nm was used to excite the samples. Fluorescence emission was collected at emission range from 450 to 600 nm. All measurements were carried out in Tris buffer, pH 7.4, at room temperature.

### 2.4. Preparation of C-apoCytc-V Expression in Stable Cell Lines

The process of viral packaging was facilitated by plating the cells on polystyrene-coated dishes. The HEK293T cells were incubated until they reached 80% confluency in about 24 h. The viral particles were prepared for the expression of C-apoCytc-V. Transfections were carried out with Fugene (Promega, Madison, WI, USA) transfection reagent (10 µL) mixed with the plasmid. After 20 min incubation at room temperature, the mixture was transferred to the cells and incubated at 37 °C, 5% CO_2_, for 15 h. The Dulbecco’s Modified Eagle’s Medium containing 10% fetal bovine serum (FBS), 100 U/mL penicillin, and 100 μg/mL streptomycin was exchanged for fresh media and incubated for 24 h. The viral particles within the supernatant were harvested by centrifugation and frozen at −20 °C until the transduction. The cells were plated and incubated at 37 °C, 5% CO_2_, until they reached 80% confluency. The vesicular stomatitis viral particles were added (200 µL) for the transduction of cells. The cells were incubated for 24 h before the media were freshly replaced. Following the selection process, they were transferred to a new plate and reached 70% confluency. The cells were then collected and stored for the imaging experiments.

### 2.5. Live Cell Imaging

To determine Cytc conformational changes in the cells, they were placed in separate wells in a 12-well nonfluorescent culture plate. An Olympus Xcellence IX-71 inverted fluorescence microscope (Tokyo, Japan) equipped with a 10× and 20× air objective, having a numerical aperture 0.3 and LED illumination system, was used for image acquisition. The cells were incubated for 12 h at 37 °C temperature and 5% CO_2_. An ANDOR-iXon3 cooled EMCCD camera (Abingdon, UK) was used to record white light illumination and Cer/Ven fluorescence images. Then, 450 nm and 512 nm light source were used for excitation of Cer and Ven, respectively. To compute the normalized FRET efficiency corrected for bleed-through values, three channel recordings of donor, acceptor, and FRET channels were applied to acquire the videos. Three filter sets were used for each channel to record the video frames. The images were acquired in four channels: (i) phase, (ii) Cer (Exc: 427 ± 10, Emis: 475 ± 20), (iii) Ven (Exc: 480 ± 25, Emis: 542 ± 27), and (iv) Cer–Ven (FRET) (Exc: 427 ± 10, Emis: 542 ± 27) for fluorescence images. Before starting the experiment, an average of 4–5 points from each well were manually scanned to determine the location of cells; 10 min/image frame rate and 75 ms exposure time were used for image acquisition. Experiments were performed in complete DMEM as described above. A total of 70 frames were recorded. Movie clips were recorded using custom stage and data acquisition software provided by the Olympus excellence system. All movie files were finally transferred to the local computer for computation of FRET signals in cells.

### 2.6. Computation of Net and Normalized FRET Signals

To determine the net and normalized FRET values, each video set was corrected with bleed-through correction factors for donor and acceptor channels [74,75,76]. The magnitude of these coefficients was determined by measuring the fluorescence emission from transiently transfected cells. Briefly, the images were recorded at Cerulean/FRET and Venus/FRET channels, respectively, after cells were transfected with Cer or Ven expressing plasmids. The emission ratio was computed and fitted to the linear function to determine the bleed-through value for Cer (D_Cer_). Similarly, fluorescence emission ratio of Ven at the FRET channels was computed to determine the bleed-through coefficient (A_Ven_). To avoid the crossover effect of Cerulean and Venus channels due to their spectral overlap, netFRET value was computed by using bleed-through coefficients (Figure S2). The netFRETs described previously were computed by using the following Equation (1):

(1) $$\mathrm{netFRET}=I_{\mathrm{FRET}}-D_{\mathrm{Cer}}{\mathrm{xI}}_{\mathrm{Cer}}-A_{\mathrm{Ven}}I_{\mathrm{Ven}}$$

where I_FRET_, I_Cerulean_, and I_Venus_ denote the background subtracted images recorded at FRET, Cerulean, and Venus channels, respectively. After background subtraction, images demonstrating the distribution of netFRET were prepared by a custom script written in MATLAB. Since the netFRET is sensitive to concentration and can vary significantly at different cells, the corrected FRET (normFRET) value was computed by using Equation (2):

(2) $$\mathrm{normFRET}=\frac{\mathrm{netFRET}}{\sqrt{I_{\mathrm{Cer}}{\mathrm{xI}}_{\mathrm{Ven}}}}$$

To determine the normFRET distribution, all traces from individual movies (n = 8) were accumulated and used to prepare histogram plots. The normFRET histograms were fitted with single or double Gaussian distribution functions. The maximum likelihood estimation (MLE) method was applied to determine the model parameters. The average normFRET values for unfolded (<E_U_>), intermediate (<E_I_>), and native state (<E_F_>) were obtained from fittings. The areas of each curve were computed and used to determine the ratio of each state. We also calculated the estimated distance between Cer and Ven (*r*_CV_) by using FRET efficiencies € and Forster radius (*R*_o_) and used the following Equation (3):

(3) $$E=\frac{1}{1+\frac{r_{CV}^{6}}{R_{o}^{6}}}$$

Forster radius (*R_o_*), by using Equation (4), was computed as 5.47 nm. Orientation factor (*κ*^2^) and refractive index values (*n*) were 1.33 and 0.66, respectively. Spectral overlap (*J*(*λ*)) of Cer and Ven was computed as 2.32 × 10^15^ M^−1^ cm^−1^ nm^−4^.

(4) $$R_{o}^{6}=8.8\times 10^{-5}\kappa^{2}n^{-4}J\left(\lambda \right)Q_{D}$$

### 2.7. Computation of FRET Signals

To determine the net or normalized FRET signal of cells and statistical analysis, we processed all video clips using a custom script written in MATLAB (R2019a, The MathWorks, Natick, MA, USA). It was composed of two main sections: (a) tracking and (b) FRET signal computation (Figure S2). To remove the pixel noise, all frames were convoluted by using a Gaussian filter with a lower bound of 3 pixels. The cells in image frames were segmented by the watershed method following image reconstruction. Their centroids and attributes were acquired from segmented images for the tracking analysis. An adaptive tracking method, described previously [73], was applied to determine the cell linkages in consecutive frames and their fluorescence signal in consecutive frames. After the fluorescence signal for all three channels were determined, we employed them to compute the normalized or netFRET signal values.

### 2.8. Statistical Tests

Statistical significance, average, and standard deviation for all measurements were computed and plotted by using the Matlab program. Unpaired *t*-test was applied to evaluate the FRET results. Unless otherwise stated, significance values were demonstrated as * *p* < 0.05, ** *p* < 0.01, and *** *p* < 0.005.

## 3. Results

### 3.1. Preparation and Characterization of C-apoCytc-V Construct

**Figure 1:**
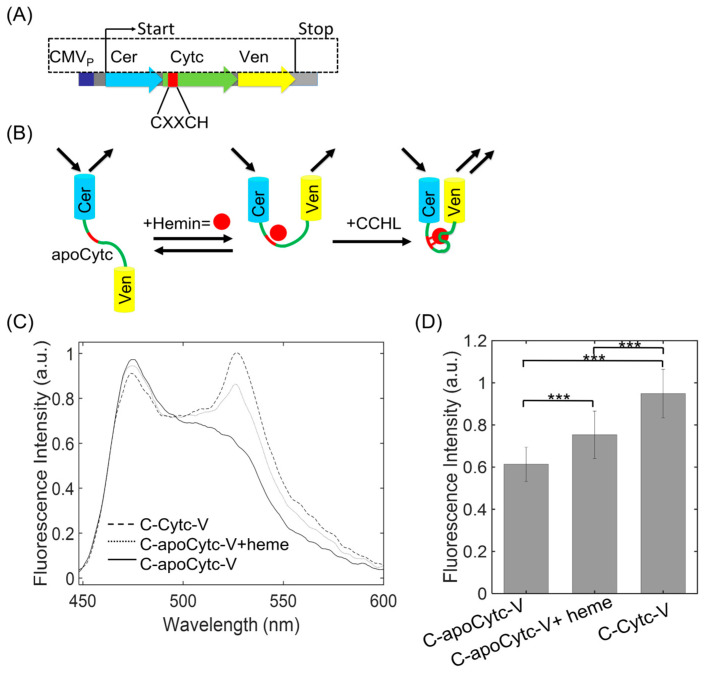
The schematic view of FRET construct that was used to study Cytc conformational changes and the fluorescence emission of C-Cytc-V and C-apoCytc-V with or without heme. (**A**) The map of the C-apoCytc-V construct. A conserved sequence of CXXCH in Cytc is shown in red. Cytc (green) attached to the N terminal of Cerulean (blue) and C terminal of Venus (yellow) was under the control of CMV promoter (violet). (**B**) The illustration demonstrated the detection of CCHL-catalyzed attachment of heme to Cytc and detection of its intermediate and folded states. In the absence of heme and CCHL, low-fluorescence emission at the Ven channel indicates the unfolded state of Cytc. In the presence of heme, noncovalent interaction induces a partial conformational change demonstrating its intermediate folding state of Cytc. In the presence of CCHL, the formation of thiol linkages between heme and Cytc stabilizes the structure and further decreases the distance between Cer and Ven; therefore, high-fluorescence signal at the acceptor channel was expected. (**C**) Emission spectra of C-apoCytc-V construct in the absence (solid line) and presence of heme (dotted line). C-holoCytc-Y demonstrates (dashed line) the highest fluorescence intensity at 527 nm, while the signal magnitude was lower for C-apoCytc-Y. The fluorescence emission was raised after the addition of heme. The emission spectrum was scanned from 450 nm to 600 nm with an excitation wavelength of 430 nm. (**D**) The comparison of fluorescence intensity changes in the absence and presence of heme. Significance values were demonstrated as *** (*p* < 0.005). The highest signal amplitude was observed for the C-Cytc-V, representing the native state of Cytc (n = 5).

We have previously shown that photochromic FRET can be used to monitor the redox state of Cytc labeled with Venus fluorescent protein [72]. The reactions of Cytc with small molecules that change its oxidation state were detected by measuring the fluorescence signal of Venus. Since photochromic FRET only allows for detection of the spectral overlap ratio, two probes in both terminals are needed to determine the conformational changes of Cytc upon interacting with heme and CCHL. We designed an FRET construct by inserting Cytc to the N and C terminals of the C17V FRET backbone, respectively (Figure 1A). We anticipated that CCHL catalyzing the heme insertion to apoCytc would induce a structural change, so the energy transfer could be higher as the distance decreases between donor and acceptor pairs. The C17V previously studied was selected to prepare the C-apoCytc-V construct with the CMV promoter site. The low baseline signal is a major advantage of this structure. Moreover, its emission signal is relatively photostable; therefore, the measurement minimally suffers from photobleaching during the data acquisition. The energy transfer from Cer to Ven can be used as an indicator to measure the distance changes between N and C terminal regions of Cytc upon binding to heme or CCHL. To detect the partially folded state of Cytc, heme can be mixed with C-apoCytc-V in the absence of CCHL, and we expect that the changes in the structure may reduce the distance between the Cer and Ven, although noncovalent association occurs between heme and Cytc (Figure 1B). The low signal amplitude was expected in the absence of both heme and CCHL due to the long distance between the FRET pairs in C-apoCytc-V. Upon addition of heme, a shift on FRET signal distribution was expected to determine the intermediate state of Cytc where noncovalent attachment of heme to Cytc backbone induces a partial recovery of its structure. Finally, CCHL catalyzes the reaction of vinyl groups in heme and cysteines in a conserved CXCCH motif that results in the formation of the native Cytc structure.

The C-apoCytc-V and C-Cytc-V constructs were initially purified from bacterial cells and characterized by a fluorescence spectrophotometer. Fluorescence emission from 450 to 600 nm was recorded to determine the energy transfer between Cer and Ven. Upon excitation at 430 nm, the emission signal for the C-apoCytc-V sample was low, indicating that a long distance was present between donor and acceptor groups. The addition of heme resulted in a higher signal amplitude at 527 nm, indicating the partial recovery of the Cytc structure (Figure 1C). The fluorescence emission was substantially increased for the C-holoCytc-V sample, indicating that the distance between Cer and Ven was low; therefore, it resulted in the highest FRET amplitude. The results demonstrated that the transition from the unfolded to native state of Cytc was detectable with the FRET method. C17V and Cer/Ven (C + V) samples were used as internal standards to evaluate the magnitude of signal changes. C17V demonstrated the highest FRET signal amplitude, while no detectable change was observed for the C + V mixture (Figure S3). The fluorescence intensity at 527 nm for all samples was compared, showing that the signal differences between C-holoCytc-V and C-apoCytc-V were statistically different (Figure 1D). We conclude that the construct was sensitive enough to study the conformational changes from unfolded to native state upon binding to heme or CCHL.

### 3.2. Characterization of Cytc Folding in the Cells by Using Timelapse Live-Cell Imaging

**Figure 2:**
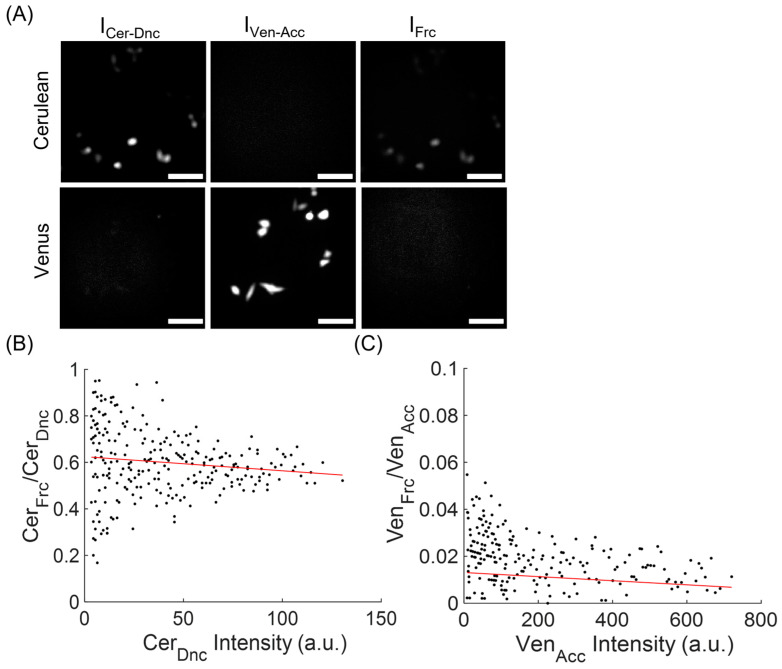
The computation of donor and acceptor bleed through coefficients that were used to compute the corrected netFRET and normFRET magnitudes. (**A**) Cer and Ven expressing cells were imaged at donor, acceptor, and FRET filter sets. (**B**) The ratio of Cer emission at FRET and donor channel is plotted against the emission at donor channel. (**C**) The ratio of Ven emission at FRET and acceptor channel is plotted against the emission at acceptor channel. The data in (**B**,**C**) are fitted to the linear function (red line) to determine the both donor and acceptor coefficients.

To determine the Cytc conformational changes in the cells, we generated the stably expressing cell line of C-apoCytc-V. The images were acquired by using the inverted fluorescence microscope equipped with three filter sets representing donor, acceptor, and FRET channels. The Cer and Ven proteins were excited at 430 nm and 470 nm, respectively. The emission signals were recorded at 480 and 530 nm filters for donor and acceptor channels, respectively. We examined the cells expressing C-apoCytc-V in the videos and computed the netFRET and normFRET amplitudes by using the signals from all three channels. To determine the coefficients used for emission crosstalk in different channels, cells expressing Cer or Ven were initially monitored, and their signal amplitude was recorded in the FRET channel. Although Ven emission signal was very low at the FRET channel, the higher fluorescence emission for Cer was observed at the FRET channel (Figure 2A). The emission intensity ratios of FRET/acceptor and FRET/donor were plotted against acceptor and donor intensity values, respectively (Figure 2B,C). After fitting each dataset to a linear function, the average values of both coefficients (donor: 0.64, acceptor: 0.012) were computed and later used for the computation of netFRET and normFRET amplitudes.

**Figure 3:**
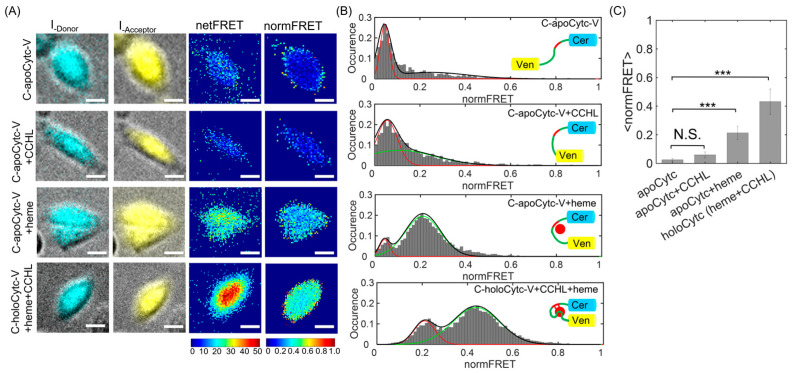
The characterization of Cytc conformational changes upon binding to heme and CCHL by using FRET assay in living cells. (**A**) Representative pseudocolor images are overlaid over the bright field images that demonstrate the expression, localization, and brightness at Cer and Ven channels (first and second columns). The panels in each row demonstrate C-apoCytc-V in the presence of heme or CCHL and C-Cytc-V, respectively. netFRET (third column) and normFRET (fourth column) amplitudes were computed after the signals at Cer and Ven channels were subtracted from images at the FRET channel. (**B**) The histograms of normFRET were obtained after dividing netFRET signal by Cer and Ven amplitudes. The data were fit with a single or double Gaussian function (red and green lines) to determine the mean FRET values. While C-apoCytc-V were fit with a single Gaussian model, heme-induced changes and C-holoCytc-V samples were best fit with a two-state Gaussian model. Unfolded, intermediate, native states were characterized by using the FRET distributions. The cumulative traces are shown in black color. The height represents the mean count of normFRET values (inset, a model of Cytc conformation states in the absence and presence of heme and CCHL). Histograms were obtained from four video datasets for each condition. Unfolded Cytc transition in the absence of heme and CCHL had a low FRET signal. After heme was added at 25 µM, noncovalent interaction induced partial conformational change in Cytc structure. Covalent attachment of heme in the presence of CCHL induced the formation of native Cytc structure, where Cer and Ven fluorophores were placed in close proximity. (**C**) Bar graph indicates the change in normFRET signal change. The significance values were shown as *** (*p* < 0.005). The elevated signal changes were attributed to the transition of Cytc from unfolded to native state (scale bar, 35 µm). N.S. means no significance.

The Cer and Ven fluorescence images overlaid on brightfield images demonstrate the stable expression of construct in the presence and absence of heme and CCHL (Figure 3A). High-fluorescence emission detected in the cytosol indicated substantial and widespread protein expression in the cells. In the absence of both heme and CCHL, representative color-coded images at donor, acceptor, and FRET channels demonstrated that both netFRET and normFRET amplitudes from C-apoCytc-V construct were undetectably low, indicating the long distance between Cer and Ven pairs (Figure 3A, first panel). The results indicated that Cytc had an unfolded structure after expressed in the cells. After the co-expression of CCHL in the absence of heme, we did not observe a significant increase in the FRET signal (Figure 3A, second panel), indicating that the association of Cytc with CCHL was weak in the absence of heme. We concluded that the low FRET signal in the absence of heme demonstrates that the cofactor was essential for initiation of Cytc folding before it associates with the CCHL protein. After an addition of 25 µM heme in the absence of CCHL, FRET signal amplitude substantially increased, demonstrating that the distance between Cer and Ven decreased due to partial folding of Cytc (Figure 3A, third panel). Finally, the highest normFRET signal was observed in the presence of both CCHL and heme due to the close distance between Cer and Ven pairs (Figure 3A, fourth panel). We conclude that Cytc formed a native-like structure when CCHL was expressed in the presence of heme. The signal amplitude observed at holoCytc was slightly lower than that observed at C17V (Figure S3), indicating a similar distance between donor and acceptor proteins in both constructs. We conclude that Cytc formed an intermediate structure in the presence of heme and required the CCHL catalyst for the native state.

To gain insight into the Cytc conformational changes, we examined the normFRET histograms computed from FRET movies. The histogram distribution for C-apoCytc-Y in the absence of heme and CCHL showed a single peak (Figure 3B, first panel). As expected, an insertion of Cytc between Cer and Ven significantly increased the distance between FRET pairs, resulting in a low-amplitude and uniform distribution of normFRET signal. The data were best fitted to single Gaussian function by using the MLE method and yielded the mean normFRET value of <E_U_> = 0.041. As a control, we compared our result with the FRET signal from the C + V sample, where the cells coexpressed Cer and Ven proteins. The normFRET signal amplitude was also low with a value of <E_C+V_> = 0.037, indicating the basal signal level in the cells (Figure S4). The mean values of both distributions were similar, suggesting that a large distance between Cer and Ven results from the unfolded structure of apoCytc. On the other hand, we were not able to distinguish if any helical secondary structure was present in the Cytc structure. To test whether the CCHL binds Cytc in the absence of heme, we examined the FRET histogram distribution (Figure 3B, second panel). The fractional change for normFRET was low, with a value of <E_U_> = 0.051 and very similar distribution to apoCytc, indicating that the association of CCHL and Cytc was weak and depended on the presence of heme. We speculated that a small shift with a second peak (<E_I_> = 0.14) may have resulted from a trace amount of free heme remaining in cells and culture solution, although we used the succinylacetone to suppress the heme metabolism before the experiment. We conclude that the expression of CCHL did not alter the FRET histogram, indicating that CCHL and Cytc had a low affinity in the absence of heme.

To determine the heme-induced conformation state of Cytc in the absence of CCHL, 25 µM heme was added to cells expressing C-apoCytc-Y. We observed a significant shift at normFRET distribution having two separated peaks at low and high amplitudes (Figure 3B, third panel). We used a two-state model that represents the unfolded and partially folded states of Cytc. The data were best fitted to a double Gaussian function and yielded the mean FRET values for corresponding populations of Cytc. A broadening peak at a high FRET ratio in the histogram resulted from the noncovalent binding of heme to Cytc. The peak values for unfolded and intermediate states were determined as <E_U_> = 0.039 and <E_I_> = 0.210, respectively. A shift was reminiscent of the heme-induced intermediate state of Cytc. We conclude that Cytc formed a compact and partially folded structure where the distance between Cer and Ven substantially decreased. The low-amplitude distribution was an indicator of the presence of remaining unfolded Cytc. By using the area of each curve, the ratio of the unfolded state to the partially folded structure was determined as A_U/I_ = 0.1. Our results suggest that the C-apoCytc-V construct was capable of undergoing conformational change upon heme interaction in the absence of CCHL.

To detect the native state of Cytc, CCHL was coexpressed with C-apoCytc-V in the presence of heme. We observed a significant shift in the normFRET distribution (Figure 3B, fourth panel). After fitting the data to a two-state model, we obtained average normFRET values of <E_I_> = 0.20 and <E_F_> = 0.41. The ratio of intermediate to native states was computed as A_I/F_ = 0.25. The peak with a small amplitude was an indicator of an intermediate structure of Cytc. The prevalence of a high FRET population results from the formation of a compact native-like tertiary structure of Cytc. We also compared our computed values with the C17V sample used as an internal standard, where the FRET changes of these constructs were studied in detail (Figure S4). A normFRET ratio of <E_C17V_> = 0.438, with a single peak indicating strong energy transfer between fluorophores due to the short distance between pairs, was consistent with previous studies. Moreover, the distance between FRET pairs was used to compute distance between Cer and Ven. To determine the distance, we used an FRET standard and plotted the FRET amplitude as a function of distance. We found that the FRET efficiency of 0.41 corresponds to a length of 5.8 nm, as compared to distance of 5.6 nm for C17V construct.

### 3.3. Time Course of Cytc Folding Change and Its Dependence on Heme Concentration

**Figure 4:**
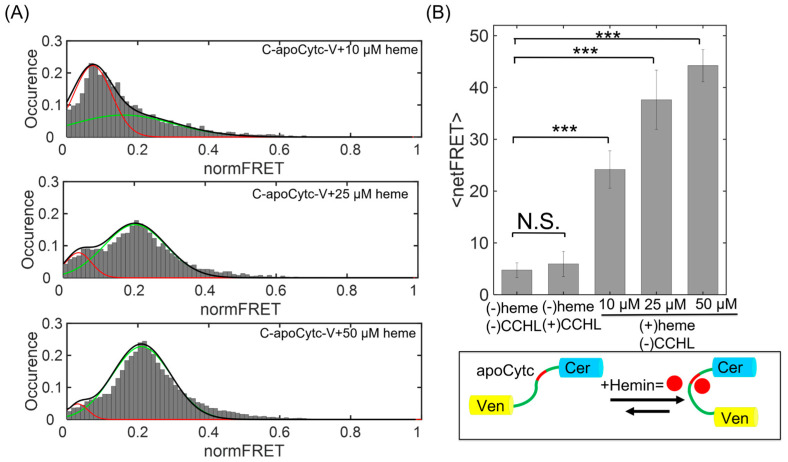
Cytc conformational change as a function of heme concentration. (**A**) normFRET distribution for C-apoCytc-C in the presence of 10, 25, and 50 µM heme. The histogram plots were obtained from n = 4 independent experiments. The histograms were fitted to single or double Gaussian functions to compute the mean values and fraction of states. The low FRET population significantly diminished after increasing heme concentration. The normFRET peaks at the mean value of 0.05 (red) and 0.2 (green) demonstrate the unfolded and partially folded states, respectively. (**B**) netFRET signal change at various heme concentrations. Significance values were demonstrated as *** (*p* < 0.005). The signal amplitude was at the highest level at 50 µM, indicating that heme strongly associates with Cytc in the absence of CCHL. N.S. means no significance.

To understand the association between Cytc and heme, we also varied the heme concentration and measured the change in FRET histogram distributions. The signal changes were evaluated at two hours after the addition of heme. At 10 µM heme, the peak value of low-intensity state <E_U_> = 0.039 was higher than second state with a mean of <E_I_> = 0.19, indicating the abundance of the unfolded state with a fraction of A_U/I_ = 9 (Figure 4A, top panel). The FRET population representing the partial folded state was significantly increased with a fraction ratio of A_U/I_ = 0.3 after the heme concentration was raised to 25 µM (Figure 4A, middle panel), indicating a strong association of heme with apoCytc. Finally, the normFRET amplitude at 50 µM heme reached the highest level (<E_U_> = 0.034 and <E_I_> = 0.22) for the partially folded state, with a fraction of A_U/I_ = 0.05 (Figure 4A, bottom panel). The FRET population obtained at low concentration was gradually decreased and shifted to a single peak centered at normFRET value of 0.21. At high heme concentrations, the partially folded state was the primary conformation of Cytc in the cells.

**Figure 5:**
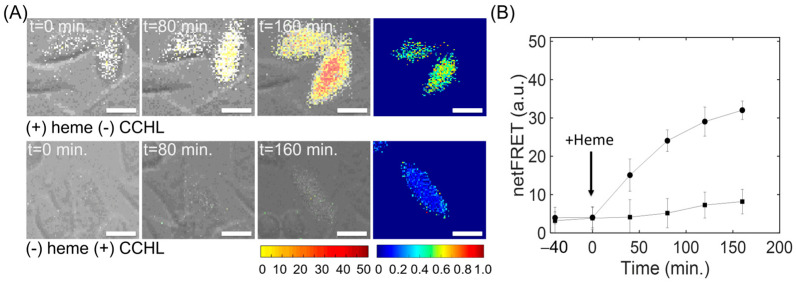
Time course of heme- or CCHL-induced conformational changes in Cytc. (**A**) Representative timelapse normFRET images of cells expressing C-apoCytc-v construct in the presence of varied heme or CCHL. The images were acquired at 0 min, 80 min, and 160 min (scale bar, 50 μm). (**B**) The time course of an average netFRET change in the presence of heme (circle) or CCHL (square). The error bars are shown for each data point. The arrow indicates the time point for the addition of heme.

To determine the time course of Cytc folding change after associating with heme, normFRET amplitude was also measured as a function of time. We observed significant changes of FRET population distribution, representing unfolded and partially folded states. FRET signal substantially increased and reached a plateau after 160 min. After the addition of 25 µM heme, we observed a significant increase in normFRET signal amplitude that was overlaid on the phase image (Figure 5A). However, C-apoCytc-V in the presence of CCHL did not demonstrate any significant change in the FRET signal. We also tested C + V as a negative control and did not observe any shift in the normFRET population after the addition of heme (Figure S4). Finally, the magnitude of netFRET signal reached a value of 30 for C-apoCytc-V in the presence of heme, while the netFRET amplitude remained below 10 in the presence of CCHL (Figure 5B). Our results suggest that the binding of heme to apoCytc induces a conformational change and does not require a CCHL catalyst for their association. The interaction of apoCytc with CCHL in the absence of heme was not detected, indicating that heme is needed for their binding.

## 4. Conclusions

In summary, this study provides a direct method to elucidate the roles of CCHL and heme on Cytc folding in living cells. We monitored the unfolded, intermediate, and native states of Cytc in the cells by using the FRET method where the N and C terminals of Cytc were labeled with Cer and Ven fluorescent proteins, respectively. In the absence of both CCHL and heme cofactor, high occurrence of a low normFRET distribution indicates the unfolded state of Cytc. We found that the noncovalent association of Cytc with heme occurs in the absence of CCHL. The formation of partially folded state was the consequence of Cytc possessing a complex with heme that may alter the orientation of helix domains. The FRET histogram indicated that the populations of unfolded and intermediate states were varied as a function of heme concentration. The changes in normFRET distribution demonstrated that the interaction of heme with unfolded Cytc drives the formation of the transition state. The FRET signal magnitude, observed at the intermediate state, was lower than the signal amplitude obtained at the native state of Cytc. We conclude that the lack of disulfide linkages catalyzed by CCHL prevented the full recovery of the Cytc folding. By contrast, CCHL weakly associates with Cytc in the absence of heme, suggesting that the complex formation strongly relies on the initial association of heme before CCHL forms a heterodimer complex with Cytc. A small change in the FRET signal was a result of remaining heme in the cytosol. In the presence of both CCHL and heme, the significant shift in the FRET histogram plot indicated that heme binds to Cytc backbone by disulfide linkage, thereby stably forming the native structure. Although we could not directly detect if CCHL was involved in the process of Cytc refolding after it catalyzed the formation of thiol bonds, we were nonetheless able to distinguish the Cytc conformational states in the presence and absence of CCHL by using the FRET method. Our results provide an insight into the role of heme- and CCHL-induced conformational changes in Cytc. Our results also demonstrate that donor and acceptor bleed-through coefficients can be used to correctly predict the normFRET magnitude for the proposed construct.

To monitor the conformational changes of Cytc, nonfluorescent approaches were used in previous studies, including absorption spectroscopy, gel filtration, FTIR, and others. Although they were extensively applied to elucidate the heme insertion mechanism, unlike our method, they cannot be used to monitor the structural changes in vivo. Our approach allows the determination of folding states of Cytc by using a Cer–Ven FRET backbone, and therefore provides a sensitive approach to study Cytc, heme, and CCHL interactions at spatial and temporal resolution. Our method can also be used to study the Cytc responses evoked by small molecules and other protein groups in living cells, therefore providing a quantitative tool to study their interactions with Cytc. It was argued that the N terminal region of CCHL was critical for binding and the formation of complex required for disulfide linkage. Indeed, the association of both proteins appears to be crucial for the formation of disulfide linkage. Our approach can be used to determine whether the CCHL initiates the rapid folding of Cytc by inducing some degree of conformational change before Cytc fully orients to the heme for sulfide linkage. Our studies currently do not distinguish if CCHL has an immediate effect on the folding of Cytc after it catalyzes the sulfide bond formation. It is crucial to determine if the transition from the intermediate to native form results from the formation of covalent linkage or CCHL, which further associates with the partial form of Cytc and affects the folding independent of its catalytic activity. It is also unclear how the linker length between Cytc and fluorescent proteins affects the ability of this probe to bind heme and CCHL. These studies are underway in our lab and will be reported in the future. Together, our study presents an optical approach that can be directly used for the characterization of the Cytc folding states in physiologically relevant conditions. The method can also be applied for understanding the conformation changes of Cytc upon interaction with other proteins such as Apaf1 or lipid membranes. The role of Cytc misfolding and associated changes in cells can be studied using fluorescence assays. Another important aspect of our method is to study the role of amino acids in Cytc and CCHL in regulating the protein folding; therefore, point mutation can be used to alter the amino acids at Cytc and investigate their roles in associating with CCHL and heme. The ability to express this probe and study Cytc maturation in different cell lines is an additional benefit of the proposed method. The methods and computational tools used for single cell tracking and FRET analysis were described in detail and can be used in other studies. There is a strong interest in developing new methods that allow for the study of the transition of proteins from denatured to native states. The FRET-based fluorescent readout provides a rapid and sensitive tool to measure dynamic conformational changes in real time [77,78]. Our methods can be expanded to study the interactions of other Cytc isoforms with small molecules and, therefore, can be directly applied to measure their effects on protein folding under physiological conditions.

## Data Availability

The data and software that support the findings of this study are available from the corresponding author upon reasonable request.

## Supplementary Materials

The following supporting information can be downloaded at: https://www.mdpi.com/article/10.3390/bios13090890/s1, Figure S1: The section of nucleotide sequence of C-Cytc-V encoding region; Figure S2: The computation of netFRET and normFRET intensity of single cells after the image acquisition and processing of raw data [73]; Figure S3: The fluorescence emission of C17V and C + V co-expressed samples; Figure S4: The evaluation of FRET efficiency by using C + V and C17V samples that are used as an internal standard in all experiments.
