# Supplementary material for: Genetically Encoded Fluorescent Probe for Detection of Heme-Induced Conformational Changes in Cytochrome c
Source: Biosensors (Basel). 2023 Sep 18;13(9):890. doi: 10.3390/bios13090890 (PMC10526477; doi:10.3390/bios13090890)
Supplement: Supplementary file 1 [file biosensors-13-00890-s001.zip › biosensors-2569664-supplementary.pdf]

biosensors

Supplementary Information

Genetically Encoded Fluorescent Probe for Detection of Heme-Induced Conformational Changes in Cytochrome c

Mehmet Yunus Genceroglu [1], Cansu Cavdar [1], Selen Manioglu [2] and Halil Bayraktar [1,*]

```
M  V  L  L  E  F  V  T  A  A  G  I  T  L  G  M  D  E  L  Y
ATGGTCCTGCTGGAGTTCGTGACCGCCGCCGGGATCACTCTCGGCATGGACGAGCTGTAC
||||||||||||||||||||||||||||||||||||||||||||||||||||||||||||
ATGGTCCTGCTGGAGTTCGTGACCGCCGCCGGGATCACTCTCGGCATGGACGAGCTGTAC

K  S  G  L  R  S  R  V  T  E  F  K  A  G  S  A  K  K  G  A
AAGTCCGGACTCAGATctcgagTGACTGAATTCAAGGCCGGTTCTGCTAAGAAAGGTGCT
||||||||||||||||||||||||||||||||||||||||||||||||||||||||||||
AAGTCCGGACTCAGATCTCGAGTGACTGAATTCAAGGCCGGTTCTGCTAAGAAAGGTGCT

T  L  F  K  T  R  C  L  Q  C  H  T  V  E  K  G  G  P  H  K
ACACTTTTCAAGACTAGATGTCTACAATGCCACACCGTGGAAAAGGGTGGCCCACATAAG
||||||||||||||||||||||||||||||||||||||||||||||||||||||||||||
ACACTTTTCAAGACTAGATGTCTACAATGCCACACCGTGGAAAAGGGTGGCCCACATAAG

V  G  P  N  L  H  G  I  F  G  R  H  S  G  Q  A  E  G  Y  S
GTTGGTCCAAACTTGCATGGTATCTTTGGCAGACACTCTGGTCAAGCTGAAGGGTATTCG
||||||||||||||||||||||||||||||||||||||||||||||||||||||||||||
GTTGGTCCAAACTTGCATGGTATCTTTGGCAGACACTCTGGTCAAGCTGAAGGGTATTCG
```

Figure S1. The section of nucleotide sequence of C-Cytc-V encoding region. The sequence of the plasmid was verified at the sequencing center of Macrogen Inc. (Amsterdam, The Netherlands). The predicted recombinant DNA construct and Macrogen sequencing results were aligned in Serial Cloner Freeware 2.6.1 for verifying the matching. (Cyan: Cerulean, Orange: XhoI restriction site, Red: Cyt c).

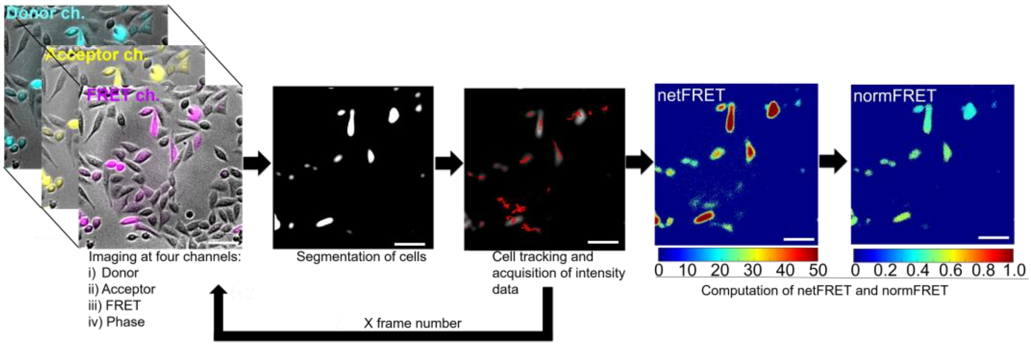

Figure S2. The computation of netFRET and normFRET intensity of single cells after the image acquisition and processing of raw data. Representative raw images of cells were acquired at donor (Cerulean), acceptor (Venus), FRET and phase channels. To determine the cell centers and connect them in consecutive frame, images were smoothed by using a gaussian filter and tracked by using a custom cell segmentation/tracking algorithm described in our previous studies [1]. After all cells were separated from background by thresholding, they were segmented with watershed method. The cell linkages in consecutive frames were determined after using single-cell adaptive tracking algorithm. The fluorescence intensity signal from each channel was extracted by using the cell

coordinates after the tracking section was completed. The netFRET was computed after the subtraction of donor and acceptor intensity values corrected for the bleed-though at both channels. At the final step, the signal intensity was normalized to determine the normFRET changes in single-cells. (scale bar, 50 μm).

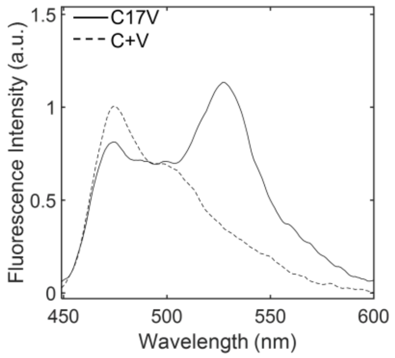

Figure S3. The fluorescence emission of C17V and C+V co-expressed samples. (A) Emission spectra of C17V construct (solid line) and C+V sample (dashed line). The fluorescence emission was higher for C17V. After excitation at 430 nm, the emission spectrum was recorded from 450 nm to 600 nm.

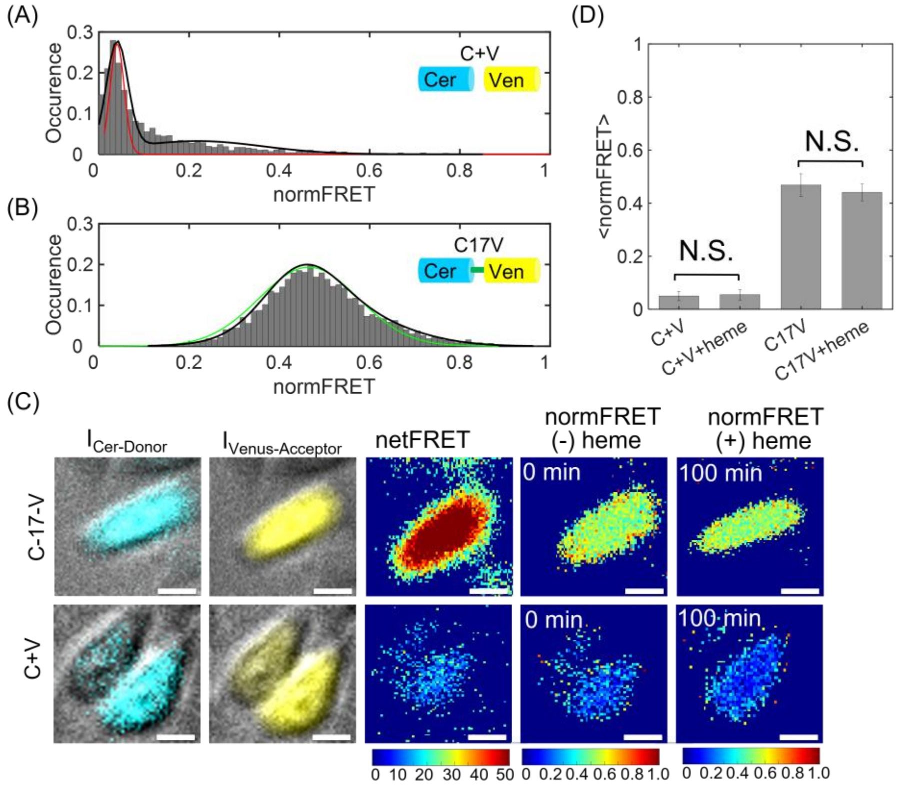

Figure S4. Evaluation of FRET efficiency by using C+V and C17V samples that are used as an internal standard in all experiments. (A) The histogram plots (gray bars) of the normFRET were computed for C+V. For C+V sample, the occurrence of low FRET with a mean value of 0.03 demonstrates the basal signal level. (B) The normFRET distribution for C-17-V sample. The result exhibits a high signal ratio for C17V due to close distance between donor and acceptor FRET pairs. All traces from videos were summed to calculate mean values. The bars were fitted to a single gaussian function (red and green lines) that were overlaid to the plot. Inset, a model of constructs expressed in the cells. (C) Representative images of cells acquired at 0 hr. and 4 hr. demonstrating the normFRET signal for C+V and C17V in the cells. The acquisition of image before and after heme addition (last two columns). As expected, the normFRET amplitude remained unchanged in the presence of 25 μM heme (D) As a control experiment, the FRET response of C17V and C+V were measured as a

function of heme. The addition of heme did not significantly change the normFRET amplitudes. (scale bar, 35 μm) .

References

1. Qureshi, H.; Ozlu, N.; Bayraktar, H. Adaptive Tracking Algorithm for Trajectory Analysis of Cells and Layer-by-Layer Assessment of Motility Dynamics. *Comp. In. Biol. Medi.* **2022**, *150*, 106193.
